# Does Paradoxical Leadership Facilitate Leaders’ Task Performance? A Perspective of Self-Regulation Theory

**DOI:** 10.3390/ijerph18073505

**Published:** 2021-03-28

**Authors:** Silu Chen, Yu Zhang, Lili Liang, Tao Shen

**Affiliations:** 1 School of Economics and Business Administration, Central China Normal University, Wuhan 430079, Hubei, China; chensilu@mail.ccnu.edu.cn (S.C.); zhang-yu@mails.ccnu.edu.cn (Y.Z.); lianglili@mails.ccnu.edu.cn (L.L.); 2 The Institute for Sustainable Development, Macau University of Science and Technology, Macau, China

**Keywords:** paradoxical leadership, job crafting, career resilience, task performance, self-regulation theory

## Abstract

As an emerging Chinese indigenous leadership style, paradoxical leadership has received considerable attention from researchers. Many studies have demonstrated the positive impact of paradoxical leadership on employees, teams, and organizations; however, there is less information on how paradoxical leaders influence their own work outcomes. On the basis of self-regulation theory, in this study, we examined the impact of paradoxical leadership on leaders’ task performance. In addition, we investigated the mediating effects of job crafting and career resilience on this relationship. Through a survey of 120 leaders and 271 of their immediate followers, our empirical analysis found the following: (1) paradoxical leadership was positively related to leaders’ task performance, (2) job crafting mediated the relationship between paradoxical leadership and leaders’ task performance, and (3) career resilience positively moderated the relationship between paradoxical leadership and job crafting, and had an indirect effect on task performance through job crafting. Our model offers novel insights into the paradoxical leadership literature and implications for improving leaders’ job crafting and task performance.

## 1. Introduction

Paradoxical leadership refers to leaders who simultaneously use two mutually opposing, interdependent, and complementary behaviors to satisfy both structural and individual needs [1]. This type of leadership is the most effective when leaders deal with organizational paradoxes related to balancing short- and long-term goals [2]. Emphasizing the coexistence of mutually independent elements, paradoxical leadership is a process that is based on personal characteristics and cognition, using a “both-and” strategy instead of an “either-or” strategy to resolve organizational paradoxes [1]. Research has documented the benefits of paradoxical leadership for employees, teams, and organizations; for example, positive impacts include the following: employees’ proficiency, adaptability, proactivity [1], voice [2], creativity [3,4], ambidexterity [5], resilience [6], in-role and innovative performance behavior [7], team perspective-taking, and innovative performance [8], as well as organizational creativity [9], ambidextrous innovation [10], and strategic agility [11].

Nevertheless, little is known about how leaders are affected by engaging in such behavior. Specifically, the literature on paradoxical leadership has mainly focused on recipients of such leadership practices; however, more recent studies have shown that there are also benefits for the leaders themselves [12,13]. Since paradoxical leadership is more abstract and less predictable than other leadership styles, to what extent this type of leader manages the contextual paradox that influences the critical success factors, so far, it has been ignored [1,14]. Meanwhile, the focus of previous studies on task performance has been on the followers rather than leaders [15,16]. In this study, we focused on leaders’ task performance, which has been considered by Organ and Paine [17] (p. 375) to be “part and parcel of the workflow that transforms inputs of energy, information, and materials into outputs in the form of goods and services to the external constituency.” Task performance is the most direct type of performance for evaluating job performance, representing an individual’s behaviors for completing the tasks specified by job responsibilities, including the quantity and quality of work expected by an organization [18]. It is usually considered to be a particular aspect of an individual’s in-role performance within an organization [19]. Specifically, previous studies have proposed that when people want to achieve good job performances, they must develop a precise understanding of their role and task requirements [20,21]. Accordingly, in contrast to previous studies that have focused on the impact of paradoxical leadership on followers’ consequences, in this study, we explore the effects of paradoxical leadership on leaders’ task performance from an actor-centric perspective.

**Figure 1:**
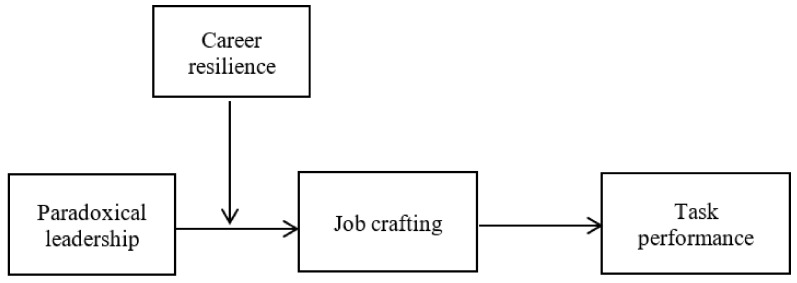
The conceptual model.

Previous studies on the effects of leadership behaviors on leaders themselves have mostly been based on resource conservation theory [13,22] or affective event theory and self-determination theory [12]; however, these studies have lacked discussions from the perspective of self-control. In order to better understand the relationship between paradoxical leadership and leaders’ task performance, we drew from self-regulation theory. Self-regulation theory is a self-control process by which people notice or anticipate differences or contradictions between themselves and their work and take the initiative to make changes [23,24]. Paradoxical leaders continuously face higher expectations that require them to use their psychological, cognitive, and emotional resources, which inevitably increases their stress [1,14]. A discrepancy between the goals and current state occurs, and negative (or positive) feedback loops develop, leading to changes in behaviors to realign goal attainment, therefore, awakening the self-regulation mechanism [25]. We thus argued that job crafting is a self-control process that is responsible for goal setting, self-improvement, and self-management behaviors, while self-regulation ensures the completion of plans, focusing on goals, inhibition of impulses, and regulation of behaviors [26,27]. Job crafting has been defined as an individual initiative that helps to improve the conflict between people and work, to enhance work adaptability, and to maintain the continuous challenge, motivation, and sense of meaningfulness in work [28]; it includes increasing work resources, increasing challenging work demands, and reducing impeding work demands [29]. Scholars have emphasized the necessity and importance of job crafting from an actor-centric perspective [30,31], whereas how leaders’ job crafting influences their own task performance is unclear. In addition, studies in developmental psychology have emphasized that resilience contains self-regulatory functions that serve to buffer the negative effects of an undesirable environment [32]. Self-regulation is an internal or transactional process that enables individuals to guide their goal-directed activities over time and across changing circumstances [33]. For example, Gardner et al. [34] regarded resilience as a relatively positive adaptation in the face of heightened risk for maladaptation and argued that such adaptation required an actor’s self-regulation. Resilience can be defined as an effective response and adaptation in the face of loss, difficulty, or adversity [35]. Career resilience extends resilience research to the career field, which is defined as an individual’s resilience to professional setbacks in a poor work environment [36,37]. Therefore, this study inferred that job crafting can transform the stress of paradoxical leadership into motivation and plays an intermediary role in the relationship between paradoxical leadership and leaders’ own task performance. Career resilience is used as a boundary condition for paradoxical leadership to influence leaders’ job crafting and task performance, thereby constructing an integrated theoretical model (Figure 1).

The key contributions of this research are as follows: First, scholars have pointed out that behaving paradoxically may tax leaders’ cognitive resources, which can cause leaders to experience psychological stress [1,14]; as such, we shifted our research focus from the influence of recipients to the sender and we explored the impact of paradoxical leadership on their job-crafting and task performance. Second, previous studies on the role of leadership practices on leaders themselves have mostly been done by considering resource conservation theory [13,22] or affective events theory and self-determination theory [12]. In this study, we investigated the impact of paradoxical leadership on leaders’ task performance based on self-regulation theory to open the above “black box,” highlighting the necessity and importance of job crafting on leaders. Finally, we took an initial step toward examining an important boundary condition, i.e., career resilience, which is considered to be the key to overcoming career stress [38]. We aimed at enriching the understanding of how career resilience explains the relationship between paradoxical leadership and leaders’ task performance.

## 2. Research Framework

### 2.1. Paradoxical Leadership and Task Performance

Paradoxical leadership is defined as the use of seemingly competing, yet inter-related, behaviors to simultaneously respond to structural and follower demands over time [1], that is, a leader adopts “both-and” behaviors that integrate and accept opposite demands simultaneously in order to gain from the intent behind the paradox [39]. Scholars insist that behaving paradoxically taxes leaders’ cognitive resources, which may cause them psychological stress and then influence their attitudes and behaviors [1,14]. In this paper, we propose a positive relationship between paradoxical leadership and leaders’ task performance from two aspects.

First, it has been shown that the nature of work stressors (i.e., challenge and hindrance) can affect individuals’ intrinsic motivation and way of thinking, and therefore, affect their work attitudes and behaviors [40]. Challenge stressors include workload, time pressure, and job complexity, which can be overcome and can have a positive impact on individual growth and development; hindrance stressors include organizational politics, role conflict, role ambiguity, and work insecurity, which are difficult to overcome and can hinder individual development and the achievement of goals [41]. In this study, we considered the pressure that paradoxical leadership brings to leaders in the form of a challenge stressor that promotes leadership progress. As a benign stressor, relevant studies have shown that challenge stressors correlate positively with task performance [40]. Therefore, we believe that paradoxical leadership requires cognitive resources and can cause pressure on leaders [1,14], and this kind of challenge stressor encourages them to achieve better task performance.

Second, according to previous studies, paradoxical leadership has four skills, i.e., cognitive complexity, confidence, conflict management, and communication [42]. In a similar vein, we explain the influence of paradoxical leadership on leaders’ task performance based on these four skills. First, cognitive complexity enables paradoxical leaders to explore contradictions to find new possibilities and reframing the existing mind-set [42]. Leaders who explore the dynamics of a contradictory tension are able to cautiously recognize new relationships and connections [43], which guide them to achieve improved task performance. Second, confident paradoxical leaders view setbacks as learning opportunities, seek to stand out from seemingly conflicting goals, and have the courage to accept challenges at every turning point [42], increasing the probability of better task performance. Furthermore, paradoxical leaders understand how to deal with difficult conflicts and how to make the best decisions [44]. They usually use creativity instead of passively responding to paradoxes and purposefully deal with competing demands to prompt themselves and their followers to question existing assumptions and seek new breakthroughs [45]. Finally, leaders need to inspire followers to accept the paradox by effectively articulating the overall vision of conflicting needs [46]. In this case, paradoxical leaders can clarify their own logic and justify their own decisions for more effective paradoxical management [42]; thus, effective communication can promote leaders’ task performance. On the basis of the above analysis, we proposed the following hypothesis:

**Hypothesis** **1** **(H1).** *Paradoxical leadership is positively associated with leaders’ task performance*.

### 2.2. Mediating Role of Job Crafting

According to the theory of self-regulation, individuals use self-regulation resources to narrow the difference between a current state and a target in order to achieve a desired state [47]. Consistent with the view of Searle and Lee [48], people usually display active behaviors to achieve a better match between work requirements and resources. This means that achieving a person-work fit is one of the purposes of people engaging in active behaviors.

Job crafting indicates the extent to which individuals change their behavior according to their abilities and needs to balance work requirements and resources [49,50], which is called spontaneous behavior change [51]. Berg et al. [52] found that the complexity and challenge of a task can stimulate an individual’s job-crafting behavior. The following self-regulating behaviors are involved in job crafting: (1) self-observation, when relevant factors motivate job crafting; (2) self-judgment, when suitable job crafting opportunities are identified; (3) self-reaction, the action of job crafting [53]. Therefore, according to this theory, paradoxical leadership, as a more complex and challenging way of leadership [1,14], encourages leaders to reshape their work, increase work resources, and realize the matching of people and work, that is, engage in job crafting. In addition, paradoxical leadership is characterized by self-confidence, which is a manifestation of self-efficacy. People become more confident when they can overcome difficulties, and they are therefore more willing to try a variety of opportunities to prove their ability [31]. In this regard, leaders with self-confidence are more willing to redesign their work and accomplish more tasks, that is to say, they are more willing to craft their jobs.

Additionally, both qualitative [52,54] and quantitative studies [29,55] have confirmed that job crafting is positively related to performance. These studies assume that job crafting can improve a leader’s task performance based on the following three reasons: first, job crafting requires modifying the number and types of tasks, the number and intensity of interactions with others, and adjusting the meaning of work according to one’s own needs to increase individual work resources [51]; second, because individuals can adjust their workload and participate in new projects, job crafting increases the demand for challenging work, which promotes personal growth and development [56]; finally, because job crafting allows individuals to change the content and scope of their work according to their own needs, it reduces obstructive work demands, as well as the pressure and burnout caused by work demands [29]. Accordingly, we suggest that paradoxical leadership activates a self-regulation mechanism and helps leaders to increase their own task performance by adjusting their work (i.e., job crafting). On the basis of the above analysis, we propose the following hypothesis:

**Hypothesis** **2** **(H2).** *Job crafting mediates the relationship between paradoxical leadership and their task performance*.

### 2.3. Moderating Role of Career Resilience

Career resilience originated from the career motivation theory put forward by London in 1983, which divides career motivation into three dimensions, i.e., career identity, career planning, and career resilience [37]. This study only used the dimension of career resilience based on Noe’s suggestion because they found that among the three components, career resilience had a more prominent predictive effect on various aspects of career behavior [57].

According to the theory of self-regulation, people’s self-regulation systems are also affected by personal factors, such as self-efficacy, personal development orientation, and self-response [58]. In this study, we suggested that career resilience has an impact on leaders’ self-regulation systems. When leaders face the pressure of contextual paradox, which continuously consumes more resources, higher career resilience gives paradoxical leaders higher self-efficacy and independence [37]. Studies have shown that when people perform well or are considered to be competent and credible, they are more likely to engage in job crafting [59,60]. In addition, independent leaders can adopt changes at work without undue restrictions [49,61] and improve job crafting. In the case that leaders’ career resilience is low, they have a lower sense of self-efficacy and higher dependence, and therefore, reduced willingness for job crafting. Furthermore, lower career resilience motivates paradoxical leaders to avoid risk [37] and choose not to change their work so that they can retain security, safety, and responsibility, resulting in a low level of job crafting [49]. On the basis of the above analysis, we proposed the following hypothesis:

**Hypothesis** **3** **(H3).** *Career resilience moderates the relationship between paradoxical leadership and job crafting. Specifically, for leaders with high career resilience, paradoxical leadership has a stronger positive impact on their job crafting, and conversely, the relationship is weaker*.

Combined with hypotheses 2 and 3, we further anticipated that the regulatory role of career resilience in the relationship between paradoxical leadership and job crafting may change the indirect effect of paradoxical leadership on leaders’ task performance through job crafting. In the case of high career resilience, the relationship between paradoxical leadership and job crafting is stronger; this means a higher sense of self-efficacy, risk-taking, and independence will regulate and strengthen the leaders’ current state of resource depletion, such as through job crafting, which improves their task performance. When career resilience is low, which means leaders have low self-efficacy and independence, and choose to avoid risks, they will engage in less job crafting, resulting in low improvement in task performance. Therefore, this research proposes the following hypothesis:

**Hypothesis** **4** **(H4).** *Career resilience moderates the indirect effect of paradoxical leadership on their own task performance through job crafting. Specifically, for leaders with high career resilience, paradoxical leadership has a stronger positive impact on their task performance through job crafting, and conversely, the relationship is weaker*.

## 3. Materials and Methods

### 3.1. Samples and Procedures

Data were collected from full-time employees and their immediate supervisors in some small and medium enterprises in Anhui, Hubei, Jiangsu, and Shanghai, in China. Details of the purpose of the study and survey instructions were clearly explained to the participants. All participants were reassured that the data were used solely for the purpose of this study and confidential. Each leader (supervisor) was responsible for evaluating the feedback from one to three followers (employees). The data were collected at two different time points, i.e., time 1 and time 2, with 1 month between the first and second time points. This time frame was similar to that used in a previous study for data processing [62] and was also selected because the temporal separation of 1 month was intended to reduce the common method variance [63] by reducing biases in participants’ retrieval and reporting of responses [64]. At the first time point, we assigned an identification (ID) to each questionnaire and matched the leaders’ (supervisors) and followers’ (employees) responses. The basic information on leaders and job crafting was evaluated by leaders (supervisors); paradoxical leadership and leader’s task performance were evaluated by followers (employees). At the second time point, we assigned the previous IDs on the questionnaires for the leaders (supervisors) to fill in to facilitate the final pairing. Career resilience was evaluated by the leaders (supervisors).

At time 1, the surveys were distributed to 181 leaders (supervisors) and 392 followers (employees). The time 1 survey was completed by 147 leaders (81.22% response rate) and 323 followers (82.40% response rate); the time 2 survey was completed by 125 leaders. After deleting blank and unmatched questionnaires, our final sample (time 2 surveys) was comprised of matched responses from 120 leaders (66.30% response) and 271 followers (69.13% response rate, using time 1 as the baseline).

In the final sample of 120 leaders, 45.83% of the participants were men. The average age was 30.58 years (SD = 7.31). A total of 12.50% of participants were under 25 years of age, 29.17% were between 25 and 35 years, 34.16% of participants were between 36 and 45 years, and 24.17% were over the age of 45. Regarding education level, the majority of participants held a bachelor’s degree or above (85.00%). With respect to job positions, both junior and middle managers accounted for 38.33%, while senior managers accounted for 23.34%. Regarding tenure, approximately 25.83% of participants had tenure of fewer than 3 years, 32.50% had between 3 and 6 years, 23.34% had between 7 and 12 years, and 18.33% had more than 12 years. About 20.00% of participants worked in educational institutions, 14.17% worked in the manufacturing industry, 16.67% worked in the financial industry, 20.83% worked in the service industry, and 28.33% worked in other types of industries.

### 3.2. Measurement Items

We followed an established back-translation procedure for translating the English scale items into Chinese [65]. First, two language specialists, fluent in both English and Chinese, independently translated the original English scale items into Chinese. Then, two other experts (not the authors) independently back-translated the Chinese scale items into English. Finally, we had a discussion with four experts to review the survey items and ensure the accuracy and validity of the translated version of the measurement items.

#### 3.2.1. Paradoxical Leadership

To measure paradoxical leadership, 22 items were developed and validated by Zhang et al. [1] (see Appendix A), which can be divided into the following five dimensions: (1) treating subordinates uniformly while allowing individualization, (2) combining self-centeredness with other-centeredness, (3) maintaining decision control while allowing autonomy, (4) enforcing work requirements while allowing flexibility, and (5) maintaining both distance and closeness. The first two dimensions were measured using five items each, and the last three dimensions were measured using four items each. Sample items are “[your leader] uses a fair approach to treat all subordinates uniformly but also treats them as individuals, shows a desire to lead but allows others to share the leadership role, controls important work issues but allows subordinates to handle details, stresses conformity in task performance but allows for exceptions, and recognizes the distinction between supervisors and subordinates but does not act superior in the leadership role.” All the items were evaluated using a five-point Likert scale, ranging from 1 (strongly disagree) to 5 (strongly agree). The scales were considered to be equivalent in Chinese culture, because Yang et al. [3] cited the scale and confirmed its reliability and validity with Chinese samples (α = 0.95). In our study, the Cronbach’s α of this scale was 0.92 (for the five dimensions it was 0.86, 0.87, 0.81, 0.78, and 0.83, respectively). The ICCs (intra class correlation) were (ICC(1) = 0.66 and ICC(2) = 0.81) and the r_wg_ (within-group interrater reliability) was 0.98, which indicated that data aggregation was appropriate.

#### 3.2.2. Task Performance

Following Methot et al. [66] (see Appendix B), task performance was measured using five items with a five-point Likert scale from 1 (strongly disagree) to 5 (strongly agree). Sample items were “[your leader] adequately completes assigned duties.” The Cronbach’s alpha value of this scale was 0.81. The ICCs were (ICC(1) = 0.52 and ICC(2) = 0.71) and the r_wg_ was 0.98, which indicated that data aggregation was appropriate.

#### 3.2.3. Job Crafting

Job crafting was measured using 15 items (α = 0.89) that were developed by Slemp and Vella-Brodrick [67] (see Appendix C). The scale measured the following three dimensions: task crafting (e.g., “introduce new approaches to improve your work”), cognitive crafting (e.g., “think about how your job gives your life purpose”), and relational crafting (e.g., “make an effort to get to know people well at work”). Respondents indicated the frequency of each crafting behavior on a five-point Likert-type scale from 1 (hardly ever) to 5 (very often).

#### 3.2.4. Career Resilience

We measured career resilience by adapting a four-item scale developed by Carson and Bedeian [68] (see Appendix D). A sample item was “the costs associated with my line of work/career field sometimes seem too great.” Respondents indicated agreement with each description on a five-point Likert-type scale from 1 (hardly ever) to 5 (very often). The Cronbach’s alpha value of this scale was 0.84.

#### 3.2.5. Control Variables

Following the recommendations for the use of theoretically potent control variables [69,70], we considered several relevant control variables, including leaders’ gender, age, education, job position, job tenure, and industries, because those factors may exert an influence on their task performance.

## 4. Results

Table 1 lists the means, standard deviations, and correlations of our variables. Paradoxical leadership was positively correlated with job crafting (*r* = 0.22, *p* < 0.05) and task performance (*r* = 0.27, *p* < 0.01), job crafting was positively correlated with task performance (*r* = 0.52, *p* < 0. 01), and career resilience was positively correlated with job crafting (*r* = 0.32, *p* < 0.01) and task performance (*r* = 0.19, *p* < 0.05).

Prior to testing our hypotheses, first, we conducted confirmatory factor analyses (CFA) using Mplus 7.0 (Muthen & Muthen, Los Angeles, CA, USA) to evaluate the distinctiveness of paradoxical leadership, job crafting, task performance, and career resilience. The results revealed that the four-factor measurement model had a good fit (Table 2), i.e., *χ*^2^ = 196.05, *χ*^2^/df = 1.74, CFI (Comparative Fit Index) = 0.90, TLI (Tucker-Lewis Index) = 0.87, RMSEA (Root Mean Square Error of Approximation) = 0.07, and SRMR (Standardized Root Mean Square Residual) = 0.07, as compared with other alternative models. Although the hypothesis model had a relatively low TLI value, as suggested by Zheng et al. [71], the observed items had significant loadings on their respective latent factors, supporting the measurements used in this study. Second, we utilized SPSS software v19.0 (IBM, Almaden, CA, USA) to conduct Herman’s single factor test on the survey data. The total variance explained by a single factor was 22.54%, indicating that the given dataset did not suffer from common method bias [64].

We used the method of hierarchical regression to test the hypotheses, which puts the control variables first. The results in Table 3 show that paradoxical leadership was positively related to task performance (model 6, *b* = 0.25, *p* < 0.01), thus supporting hypothesis 1. In addition, paradoxical leadership was positively related to job crafting (model 2, *b* = 0.23, *p* < 0.05) and job crafting was also positively related to task performance (model 7, *b* = 0.39, *p* < 0.001). When paradoxical leadership and job crafting were combined for predicting task performance, the coefficient of job crafting was still significant (model 8, *b* = 0.35, *p* < 0.001) and the coefficient of paradoxical leadership decreased (model 8, *b* = 0.17, *p* < 0.05), thus supporting hypothesis 2.

To further verify hypothesis 2, we used the bootstrap method with 5000 samples with a 95% confidence interval, as proposed by Preacher and Hayes [72]. The results are shown in Table 4. The confidence interval of the indirect effect of paradoxical leadership on task performance through job crafting was 0.08 (95% CI: 0.01, 0.21), indicating the mediating role of job crafting; the confidence interval of the direct effect of paradoxical leadership on task performance was 0.17 (95% CI: 0.02, 0.32), indicating that job crafting played a partial role of a mediator, thus further verifying hypothesis 2.

**Figure 2:**
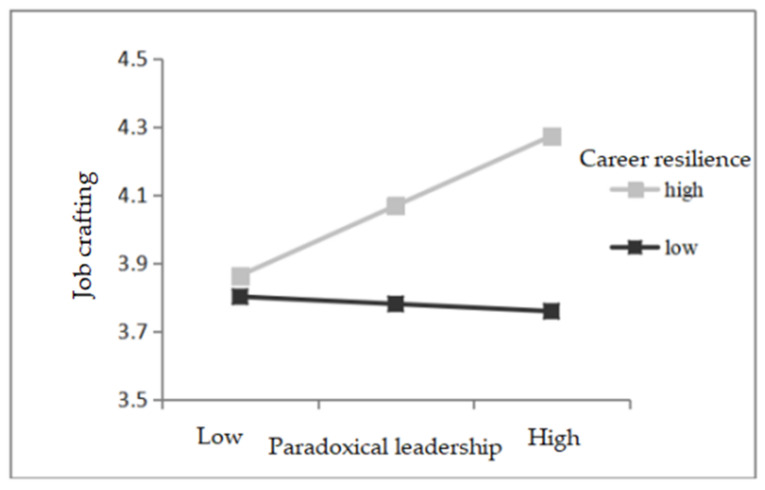
The moderating effect of career resilience between paradoxical leadership and job crafting.

Hypothesis 3 proposed that the career resilience of leaders moderates the positive relationship between paradoxical leadership and job crafting. To test hypothesis 3, first, we put the control variables in the regression equation; second, the paradoxical leader and career resilience were put into the equation; thirdly, the interaction term of the paradoxical leadership and the career resilience (to prevent multicollinearity, the interaction term of the independent variable and the moderating variable was mean-centered) were put into the equation (as shown in Table 3). The interaction term of paradoxical leadership and career resilience was positively related to job crafting (model 4, *b* = 0.36, *p* < 0.05), supporting hypothesis 3. According to Aiken and West’s [73] suggestion, the effect of the interaction was plotted and the high/low level of career resilience was divided based on the mean plus or minus one standard deviation. As shown in Figure 2, the results of the simple slope test showed that for leaders with high career resilience, paradoxical leadership had a relatively stronger positive effect on job crafting; for leaders with low career resilience, the effect of paradoxical leadership on job crafting was not significant (simple slope for high resilience = 0.44, *t* = 2.88, *p <* 0.001; simple slope for low resilience = −0.05, *t* = −0.33, ns), thus further verifying hypothesis 3.

Finally, we used model 7 of the process to generate bootstrap confidence intervals for the conditional indirect effect of paradoxical leadership on their own task performance via job crafting (see Table 5). The result demonstrated that paradoxical leadership with a high level of career resilience had a positive indirect impact on leaders’ task performance (*b* = 0.16, bias-corrected (95% CI: 0.03, 0.31)). While paradoxical leadership with a low level of career resilience, the indirect effect was not significant (*b* = −0.02, bias-corrected (95% CI: −0.11, 0.09)). Furthermore, the index of the moderated mediation was 0.13 (95% CI: 0.01, 0.26), indicating that the indirect effect of paradoxical leadership on task performance through job crafting was moderated by career resilience [74], thus supporting hypothesis 4.

## 5. Discussion

On the basis of self-regulation theory, we developed and tested a model to explain how and when engaging in paradoxical leadership affects leaders’ job crafting and task performance. The results of a questionnaire survey of 120 leaders and 271 followers from small- and medium-sized Chinese enterprises found that paradoxical leadership promoted leaders’ job crafting, thereby improving their own task performance. Moreover, the effect of paradoxical leadership on leaders’ job crafting depended on the leader’s career resilience. Specifically, the positive effect of paradoxical leadership on leaders’ job crafting was stronger among leaders with higher career resilience. In addition, career resilience had a positive moderating effect on paradoxical leadership for improving task performance through job crafting, i.e., as career resilience increased, the indirect effect of paradoxical leadership on task performance through job crafting increased.

### 5.1. Theoretical Contributions

Our research makes several key theoretical contributions. First, we contribute to the literature on paradoxical leadership by taking an actor-centric perspective and focusing on the intrapersonal consequences of paradoxical leaders and their own task performance. While previous studies have established that paradoxical leadership has an impact on followers [1,2,3,4,5,6,7], the impact on leaders who engage in paradoxical leadership behaviors has been largely overlooked since working through paradoxes is an effortful, multi-stepped process [46]. Behaving paradoxically may tax leaders’ cognitive resources, which can result in psychological stress for leaders, and therefore, possibly influence their attitudes and behaviors [1,14]. Our study provides additional understanding of the consequences of paradoxical leadership and examines its potential influence on leaders themselves.

Second, from the perspective of self-regulation theory, in this study, we explored the psychological mechanism of paradoxical leadership on leaders’ task performance, which we considered to be a self-regulation process. Although some previous studies have explored the effects of certain leadership styles, such as transformational and abusive styles, on leaders themselves, most studies have been based on the theory of resource conservation [13,22] or affective events theory and self-determination theory [12] and ignored the self-regulation mechanism of job crafting between leaders and their performance. In addition, as compared with the vast amount of research on employees’ job crafting, there is limited information about how job crafting contributes to leaders’ outcomes. In response to the suggestion that managers engage in job crafting because of its positive influence on their performance [30,31], our study introduced job crafting as the path of action, providing a more appropriate perspective for understanding the impact of paradoxical leaders on their own task performance.

Finally, this study has important theoretical constructive significance for a deeper understanding of the relationship between paradoxical leaders and their own task performance. As the internal structure of arousing career decision-making and behavior, career resilience can enable individuals to work hard and have the courage to overcome various career barriers, such as job switching and job stress [75]. However, few studies have used it as boundary conditions for the influence mechanism of leadership behaviors on job crafting. Therefore, this study addressed this shortcoming and found that career resilience is an important contingency factor affecting leaders’ behaviors.

### 5.2. Practical Implications

Our research findings provide several important managerial implications. First, organizations need to acknowledge the limitations of their existing leadership styles in order to confront the contradictory requirements in today’s dynamic internal environment. Organizations are also encouraged to popularize and train leaders in paradoxical knowledge to strengthen their ability to effectively solve the problem of paradoxes. Today, leaders should be encouraged to change the previous thinking of “one or the other” and cultivate their own integration and paradoxical thinking to deal with the increasing uncertainties.

Second, our findings suggest that job crafting is a useful tool for enhancing the task performance of leaders. When leaders implement paradoxical leadership practices, they inevitably face a large loss of resources and energy. The organization should give sufficient autonomy and support to enable leaders to satisfy their needs. It enables leaders to adjust their work according to their own needs and interests, and therefore, promotes task performance through job crafting. Our study shows that paradoxical leadership is conducive to leaders’ task performance through the intermediary mechanism of job crafting, which provides an effective way for paradoxical leaders to improve their task performance under stress.

Finally, organizations should pay more attention to leaders with low career resilience. Career resilience is a form of adaptability that involves continuous learning and the acceptance of new things. Organizations should provide low-career-resilience leaders with more opportunities to learn and accept new things to cultivate their career resilience such that they can still adopt a positive response when facing the pressure brought about by resource consumption.

### 5.3. Limitations

This research also has some limitations and needs to be further deepened. First, this is a cross-sectional study, which makes it difficult to draw conclusions about causality. Future research should employ a longitudinal study to show the cause-and-effect relationship between the variables of this study to enhance the strength of the causality argumentation. In addition, future research should be supplemented by more differentiated scenario designs for experimental data measurement to investigate whether the hypothesis in this article is still supported in richer scenarios.

Second, we used a convenient sample, where the participants were approached using the snowball sampling method. Therefore, our sample represented small- and medium-sized enterprises and caution should be taken when generalizing our findings to different enterprise sizes. Therefore, future research should expand the sample to large enterprises. In addition, since we collected data on leaders and followers from Chinese organizations, our results may not hold validity in other cultures. Therefore, we suggest future studies conduct a cross-country examination of paradoxical leadership and leaders’ attitudes and behaviors in both Eastern and Western cultures.

Finally, although this study confirmed the role of career resilience in paradoxical leadership and job crafting from the perspective of self-regulation theory, it did not consider other contextual factors (such as the task context). Therefore, future research on the moderating effects of other situational factors that may affect leaders’ job crafting and their consequences would enhance the reliability of our conclusions.

## 6. Conclusions

This study represents an initial attempt to explore the impacts of paradoxical leadership on leaders rather than the recipients of such behaviors. In particular, we highlighted the potential benefits of such behavior for leaders, which included job crafting and task performance. These beneficial effects were further enhanced by career resilience. Awareness of the benefits of paradoxical leadership for leaders could be leveraged to effectively increase their task performance through job crafting. We hope that our study motivates scholars’ interest to further explore leadership practices that trigger leaders’ psychological mechanisms and subsequent behaviors.

## Figures and Tables

**Table 1:** Descriptive statistics and correlations.

Variable	1	2	3	4	5	6	7	8	9	10
1. Gender	-									
2. Age	−0.17	-								
3. Education	−0.08	0.01	-							
4. Job rank	−0.24 **	0.66 **	0.15	-						
5. Job tenure	−0.17	0.79 **	−0.08	0.67 **	-					
6. Nature of job	−0.01	−0.11	−0.18	−0.05	0.04	-				
7. Paradoxical leadership	−0.22 *	0.28 **	−0.17	0.22 *	0.18 *	−0.04	-			
8. Job crafting	−0.12	0.16	0.10	0.11	0.17	−0.26 **	0.22 *	-		
9. Career resilience	0.10	0.06	−0.10	−0.01	0.12	−0.03	0.14	0.32 **	-	
10. Task performance	0.10	0.17	0.13	0.18 *	0.13	−0.21 *	0.27 **	0.52 **	0.19 *	-
Mean	1.54	2.78	4.21	1.85	2.73	3.50	3.96	3.94	3.21	4.40
SD	0.50	1.10	0.97	0.77	1.63	1.91	0.47	0.51	0.67	0.39

Notes: N = 120, * *p* < 0.05 and ** *p* < 0.01.

**Table 2:** Confirmatory factor analysis results.

Model	*χ* ^2^	*χ*^2^/df	CFI	TLI	RMSEA	SRMR
Four-factor model	196.05	1.74	0.90	0.87	0.07	0.07
Three-factor model 1	261.18	2.25	0.82	0.79	0.10	0.08
Three-factor model 2	290.84	2.51	0.78	0.74	0.11	0.12
Three-factor model 3	311.76	2.69	0.75	0.71	0.12	0.13

Notes: Three-factor model 1—paradoxical leadership, job crafting + task performance, and career resilience; three-factor model 2—paradoxical leadership, career resilience + job crafting, and task performance; three-factor model 3—paradoxical leadership + job crafting, task performance, and career resilience; four-factor model—paradoxical leadership, job crafting, career resilience, and task performance. CFI: Comparative Fit Index, TLI: Tucker-Lewis Index, RMSEA: Root Mean Square Error of Approximation, SRMR: Standardized Root Mean Square Residual.

**Table 3:** Analysis of the mediation and moderating effects.

Variable	Job Crafting				Task Performance			
	Model 1	Model 2	Model 3	Model 4	Model 5	Model 6	Model 7	Model 8
Gender	−0.10	−0.06	−0.10	−0.11	0.12	0.16 *	0.16 *	0.18 ***
Age	−0.01	−0.05	−0.03	0.002	0.02	−0.01	0.03	0.003
Education	0.04	0.06	0.06	0.07	0.04	0.07	0.02	0.04
Job rank	−0.06	−0.08	−0.05	−0.04	0.07	0.05	0.09	0.07
Job tenure	0.08	0.09	0.07	0.04	0.01	0.02	−0.02	−0.01
Nature of job	−0.07 **	−0.07 **	−0.07 **	−0.06 *	−0.04 ^†^	−0.04	−0.01	−0.01
Paradoxical leadership		0.23 *	0.17 ^†^	0.19 ^†^		0.25 **		0.17 *
Job crafting							0.39 ***	0.35 ***
Career resilience			0.22 **	0.21 ** 0.36 *				
Paradoxical leadership × career resilience								
∆*R*^2^	0.12	0.04	0.08	0.04	0.11	0.08	0.23	0.19
*F*	2.48 *	2.92 *	4.13 ***	4.39 ***	2.20 *	3.56 **	8.06 ***	8.09 ***

Notes: N = 120; ^†^ *p* < 0.1, * *p* < 0.05, ** *p* < 0.01, and *** *p* < 0.001 (the result is an unstandardized regression coefficient).

**Table 4:** Test of the mediating effect of job crafting.

Effects	Estimate	95% Confidence Interval
Total effect Direct effect (paradoxical leadership→task performance)	0.25	(0.11, 0.39)
	0.17	(0.02, 0.32)
Indirect effect (paradoxical leadership→job crafting→task performance)	0.08	(0.01, 0.21)

**Table 5:** Conditional indirect effect test.

Mediator	Indirect Effect	95% Confidence Interval
Low career resilience	−0.02	(−0.11, 0.09)
High career resilience	0.16	(0.03, 0.31)
Index of moderated mediation	0.13	(0.01, 0.26)

## Data Availability

The data presented in this study are available on request from the corresponding author.

## Appendix A. Items Used to Measure Paradoxical Leadership

*Treating subordinates uniformly while allowing individualization*

1. Uses a fair approach to treat all subordinates uniformly but also treats them as individuals.
2. Puts all subordinates on an equal footing but considers their individual traits or personalities.
3. Communicates with subordinates uniformly without discrimination but varies his/her communication style depending on their individual characteristics or needs.
4. Manages subordinates uniformly but considers their individualized needs.
5. Assigns equal workloads but considers individual strengths and capabilities to handle different tasks.

*Combining self-centeredness with other-centeredness*

6. Shows a desire to lead but allows others to share the leadership role.
7. Likes to be the center of attention but allows others to share the spotlight as well.
8. Insists on getting respect but also shows respect toward others.
9. Has a high self-opinion but shows awareness of personal imperfection and the value of other people.
10. Is confident regarding personal ideas and beliefs, but acknowledges that he or she can learn from others.

*Maintaining decision control while allowing for autonomy*

11. Controls important work issues but lets subordinates handle details.
12. Makes final decisions for subordinates but lets subordinates control specific work processes.
13. Makes decisions about big issues but delegates lesser issues to subordinates.
14. Maintains overall control but gives subordinates appropriate autonomy.

*Enforcing work requirements while allowing flexibility*

15. Stresses conformity in task performance but allows for exceptions.
16. Clarifies work requirements but does not micromanage work.
17. Is highly demanding regarding work performance but is not hypercritical.
18. Has high requirements but allows subordinates to make mistakes.

*Maintaining both distance and closeness*

19. Recognizes the distinction between supervisors and subordinates but does not act superior in the leadership role.
20. Keeps distance from subordinates but does not aloof.
21. Maintains position differences but upholds subordinates’dignity.
22. Maintains distance from subordinates at work but is also amiable toward them.

Notes: The above 22 items were from Zhang et al. [1].

## Appendix B. Items Used to Measure Task Performance

1. Adequately completes assigned duties.
2. Fulfills responsibilities specified in his/her job description.
3. Performs tasks that are expected of him/her.
4. Meets formal performance requirements of the job.
5. Engages in activities that will directly affect his/her performance evaluations.

Notes: The above five items were from Methot et al. [66].

## Appendix C. Items Used to Measure Job Crafting

*Task crafting*

1. Introduce new approaches to improve your work.
2. Change the scope or types of tasks that you complete at work.
3. Introduce new work tasks that you think better suit your skills or interests.
4. Choose to take on additional tasks at work.
5. Give preference to work tasks that suit your skills or interests.

*Cognitive crafting*

6. Think about how your job gives your life purpose.
7. Remind yourself about the significance your work has for the success of the organization.
8. Remind yourself of the importance of your work for the broader community.
9. Think about the ways in which your work positively impacts your life.
10. Reflect on the role your job has for your overall well-being.

*Relational crafting*

11. Make an effort to get to know people well at work.
12. Organize or attend work-related social functions.
13. Organize special events in the workplace (e.g., celebrating a co-worker’s birthday).
14. Choose to mentor new employees (officially or unofficially).
15. Make friends with people at work who have similar skills or interests.

Notes: The above 15 items were from Slemp and Vella-Brodrick [67].

## Appendix D. Items Used to Measure Career Resilience

1. The costs associated with my line of work/career field sometimes seem too great.
2. Given the problems I encounter in this line of work/career field, I sometimes wonder if I get enough out of it.
3. Given the problems in this line of work/career field, I sometimes wonder if the personal burden is worth it.
4. The discomforts associated with my line of work/career field sometimes seem too great.

Notes: The above four items were from Carson and Bedeian [68].
